# Supplementary material for: Soil Type Affects Organic Acid Production and Phosphorus Solubilization Efficiency Mediated by Several Native Fungal Strains from Mexico
Source: Microorganisms. 2020 Sep 2;8(9):1337. doi: 10.3390/microorganisms8091337 (PMC7565533; doi:10.3390/microorganisms8091337)
Supplement: Supplementary file 1 [file microorganisms-08-01337-s001.pdf]

Supplementary material

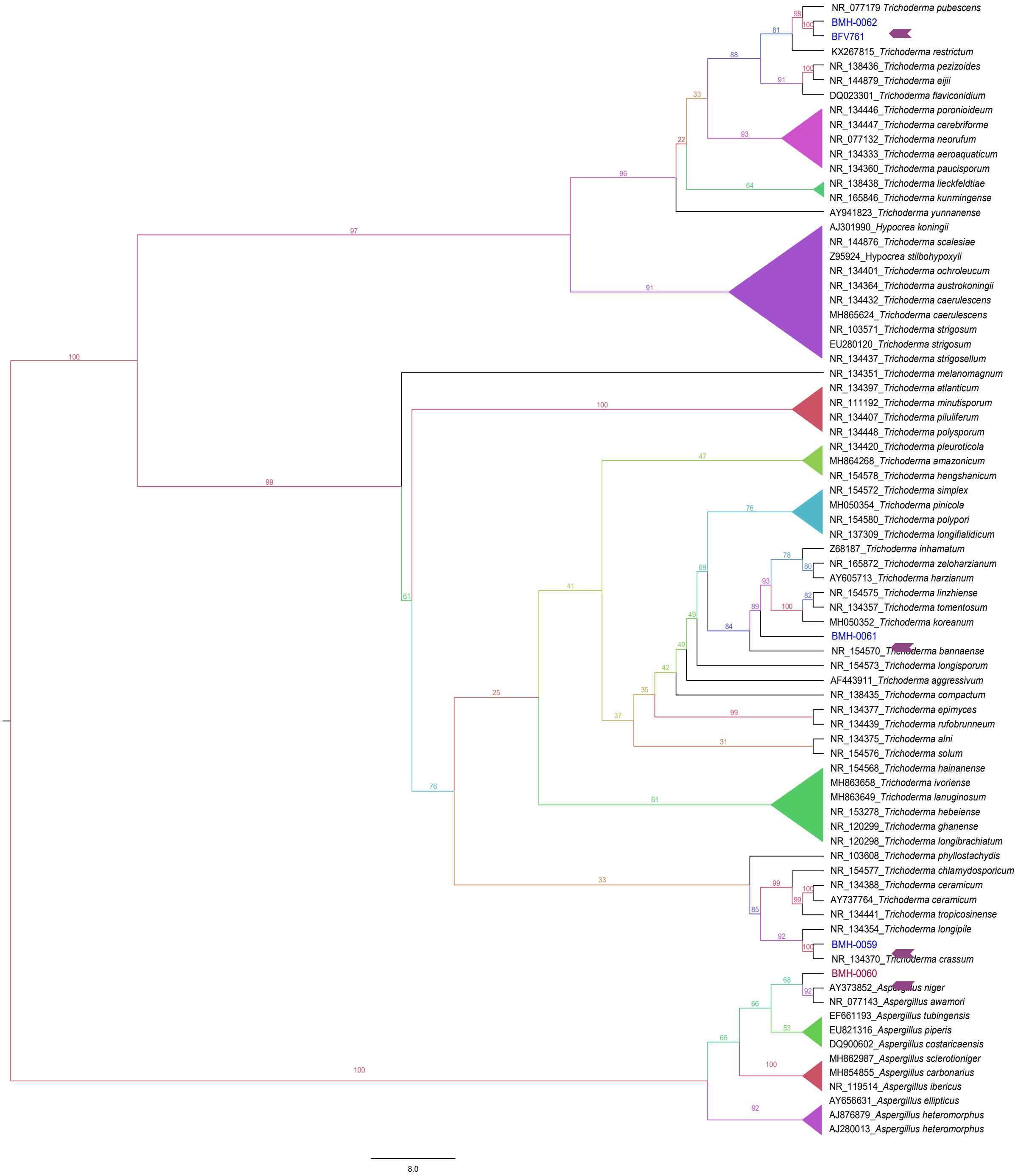

Figure S1. Phylogenetic tree generated by FastTree analysis using a MAFFT alignment of ITS nucleotide sequences obtained from the type material. The tree includes isolates related to BMH-0059, identified as *T. crassum*; BMH-0060 identified as *A. awamori*; BMH-0061 identified as *Trichoderma* sp.; and BMH-0062 identified as *T. pubescens*. Bootstrap values (> 50%) are labeled in color on the branch nodes. Color labeled triangles represent subtrees drawn as cartoon.

Table S1. Classification, location and some physico-chemical properties of the studied soils.

Georeferencing and physico-chemical characteristics of the studied soils			
**Soil**	Alfisol	Andisol	Vertisol
**Country**	Mexico	Mexico	Mexico
**State**	Morelos	Morelos	Morelos
**Municipality**	Cuernavaca	Huitzilac	Temixco
**Locality**	Chamilpa	Tres Marías	Cuentepec
**Coordinates**	18º58′35′′N 99º13′36′′W, 1824 masl	19º02′18′′N 99º15′11′′W; 2803 masl	18º51′36′′N 99º19′29′′W; 1480 masl
**Taxonomic order**	Alfisol (Haplustalf)	Andisol (Melanudand)	Vertisol (Aquerts)
**Sand (%)**	38	92	56
**Silt (%)**	30	4	24
**Clay (%)**	32	4	20
**Texture (Bouyoucos)**	Far	A	FarA
**pH (w, 1:2.5)**	7.0	5.7	4.9
**M.O. (Walkley-Black) (%)**	5.35	11	3.0
**Ca (Ammonium acetate 1M) ($cmol_c\ kg^{-1}$)**	19.9	1.7	3.0
**Mg (Ammonium acetate 1M) ($cmol_c\ kg^{-1}$)**	7.7	0.1	1.7
**K (Ammonium acetate1M) ($cmol_c\ kg^{-1}$)**	0.28	0.05	0.16
**Na (Ammonium acetate1M) ($cmol_c\ kg^{-1}$)**	0.30	0.0	0.0
**Al (KCl 1M) ($cmol_c\ kg^{-1}$)**	0.0	0.0	1.6
**CIC effective ($cmol_c\ kg^{-1}$)**	28.2	1.85	6.5
**P Bray II ($mg\ kg^{-1}$)**	32	1	34
**S (Calcium phosphate 0.008 M) ($mg\ kg^{-1}$)**	12	6	2
**Fe (Olsen-EDTA) ($mg\ kg^{-1}$)**	22	64	42
**Mn (Olsen-EDTA) ($mg\ kg^{-1}$)**	4	1	53
**Cu (Olsen-EDTA) ($mg\ kg^{-1}$)**	2	1	2
**Zn (Olsen-EDTA) ($mg\ kg^{-1}$)**	3	1	3
**B Hot water) ($mg\ kg^{-1}$)**	0.6	0.1	0.14
**NO_3 (Aluminum sulfate 0.025 M) ($mg\ kg^{-1}$)**	45	3	1
**NH_4 (KCl 1M) ($mg\ kg^{-1}$)**	11	18	38

Table S2. Detection of the type and concentration of OAs in different soils with different strains. The values are the mean of the concentration of acids (µg/mL) ± SE found in the samples of four different soil samples in their interaction with microorganisms A, B, C and D (n = 4). Grey shadowed squares depict the condition in which the highest value for that particular acid was found. "Total" at the bottom of the table refers to the average of the means of the data population.

Soil	Microorganism	Pyruvic µg/mL	Fumaric µg/mL	Tartaric µg/mL	Succinic µg/mL	Malic µg/mL	Oxalic µg/mL	Citric µg/mL	Concentration P mg/L
**Andisol**	**BMH-0059**	**0.076** ± 0.015	**0.002** ± 0.000	**0.170** ± 0.057	**0.038** ± 0.022	**0.017** ± 0.016	**0.003** ± 0.002	**0.019** ± 0.007	**4.605** ± 0.072
	**BMH-0060**	**0.236** ± 0.081	**0.002** ± 0.000	**0.070** ± 0.040	**0.034** ± 0.019	**0.354** ± 0.240	**0.057** ± 0.020	**0.033** ± 0.004	**22.072** ± 2.73
	**BMH-0061**	**0.422** ± 0.142	**0.002** ± 0.001	**0.496** ± 0.049	**0.002** ± 0.001	**0.058** ± 0.043	**0.006** ± 0.001	**0.003** ± 0.002	**29.187** ± 0.400
	**BMH-0062**	**0.122** ± 0.070	**0.014** ± 0.001	**0.404** ± 0.238	**0.124** ± 0.041	**0.007** ± 0.014	**0.005** ± 0.000	0.000	**35.217** ± 0.0800
**Alfisol**	**BMH-0059**	**0.021** ± 0.001	**0.004** ± 0.000	**0.003** ± 0.000	**0.054** ± 0.008	**0.003** ± 0.002	**0.002** ± 0.001	**0.001** ± 0.000	**2.143** ± 0.011
	**BMH-0060**	**4.126** ± 0.026	**0.028** ± 0.003	0.000	**0.000** ± 0.000	**0.006** ± 0.001	**0.032** ± 0.014	**0.393** ± 0.044	**24.185** ± 0.385
	**BMH-0061**	**0.027** ± 0.026	**0.007** ± 0.000	**0.014** ± 0.008	**0.002** ± 0.000	**0.003** ± 0.003	**0.008** ± 0.001	**0.010** ± 0.001	**34.652** ± 0.255
	**BMH-0062**	**0.056** ± 0.032	**0.037** ± 0.008	**3.126** ± 0.221	**0.010** ± 0.005	**0.002** ± 0.003	**0.023** ± 0.002	**0.020** ± 0.006	**27.693** ± 0.714
**Vertisol**	**BMH-0059**	**0.141** ± 0.023	**0.003** ± 0.000	**0.059** ± 0.003	**0.014** ± 0.004	**0.003** ± 0.002	**0.007** ± 0.000	**0.050** ± 0.024	**8.689** ± 0.237
	**BMH-0060**	**0.170** ± 0.011	**0.004** ± 0.001	**5.765** ± 0.219	**0.013** ± 0.004	**0.005** ± 0.004	**0.014** ± 0.002	**0.031** ± 0.001	**31.215** ± 0.351
	**BMH-0061**	**0.063** ± 0.008	**0.010** ± 0.000	**0.032** ± 0.019	**0.028** ± 0.003	**0.014** ± 0.003	**0.023** ± 0.001	**0.005** ± 0.000	**34.240** ± 0.209
	**BMH-0062**	**0.049** ± 0.028	**0.052** ± 0.000	**1.834** ± 0.004	0.000	**0.024** ± 0.006	**0.062** ± 0.009	**0.005** ± 0.001	**36.369** ± 0.711
**Without Soil**	**BMH-0059**	0.000	**0.001** ± 0.000	0.000	**0.092** ± 0.002	0.000	**0.171** ± 0.001	0.000	**12.191** ± 0.569
	**BMH-0060**	0.000	**0.001** ± 0.000	0.000	**0.002** ± 0.000	**0.003** ± 0.001	**0.022** ± 0.004	0.000	**31.188** ± 0.240
	**BMH-0061**	0.000	**0.001** ± 0.000	0.000	0.000	**0.004** ± 0.003	**0.050** ± 0.032	0.000	**40.615** ± 0.883
	**BMH-0062**	0.000	**0.002** ± 0.001	0.000	**0.036** ± 0.012	**0.032** ± 0.046	**0.005** ± 0.001	0.000	**37.505** ± 0.350
**TOTAL**		**0.344**	**0.011** ± **0.001**	**0.748** ± **0.195**	**0.028** ± **0.005**	**0.033** ± **0.101**	**0.030** ± **0.005**	**0.036**	**25.735** ± 1.507

Table S3. Coefficient of correlation and percentage of contribution of each variable (OAs) to the total length of the PC1 and PC2. In bold those OAs whose percentage with contribution > 10% to the eigen value length.

Acid	Correlation PC 1	Variable contribution PC1	Correlation PC 2	Variable contribution PC2
**Pyruvic**	**-0.93**	**39.83**	-0.29	5.86
**Fumaric**	-0.51	**12.01**	0.43	**13.08**
**Tartaric**	-0.06	0.17	**0.75**	**39.27**
**Succinic**	0.40	7.49	-0.45	**13.87**
**Malic**	0.13	0.75	-0.41	**11.59**
**Oxalic**	0.08	0.32	-0.39	**10.73**
**Citric**	**-0.93**	**39.43**	-0.28	5.59

Table S4: GLM table of the effect of soil type factors and microorganism on phosphorus, having the PC 1 and PC2 as covariates.

Source of variation	SS	Degr. Of	MS	F	P
**Intercept**	42387.51	1	42387.51	55341.55	**0.000000**
**Soil**	421.23	3	140.41	183.32	**0.000000**
**Microorganism**	4414.77	3	1471.59	1921.32	**0.000000**
**"PC1"**	0.31	1	0.31	0.40	0.529736
**"PC2"**	0.01	1	0.01	0.02	0.893958
**Soil*Microorganism**	258.64	9	28.74	37.52	**0.000000**
**Error**	35.23	46	0.77		**0.000000**
